# Shrinking Lung Syndrome: A Rare Pulmonary Complication of Systemic Lupus Erythematosus

**DOI:** 10.7759/cureus.63990

**Published:** 2024-07-06

**Authors:** Kejal Shah, Hema Kondakindi, Joud Enabi, Srikanth Mukkera

**Affiliations:** 1 Internal Medicine, Texas Tech University Health Sciences Center, Odessa, USA; 2 Rheumatology, Texas Tech University Health Sciences Center, Odessa, USA

**Keywords:** systemic lupus erythematous, diaphragm dysfunction, restrictive lung disease, diaphragmatic elevation, shrinking lung syndrome

## Abstract

Shrinking lung syndrome (SLS) is a rare pulmonary complication primarily associated with autoimmune diseases such as systemic lupus erythematosus (SLE). A 38-year-old female recently diagnosed with SLE on hydroxychloroquine, prednisone, and methotrexate presented with a one-week history of progressive shortness of breath, non-productive cough, and pleuritic chest pain. She was afebrile with adequate oxygen saturation. Examination revealed a few fine crackles in the lung fields. Laboratory results showed pancytopenia.

Initial treatment included broad-spectrum antibiotics and intravenous methylprednisolone for a suspected lupus flare. Cultures and tests for infections, including tuberculosis, were negative. Imaging revealed bilateral airspace disease with no pulmonary embolism. Autoimmune workup showed high antinuclear antibodies, positive anticardiolipin antibody, ribonucleoprotein, and anti-Smith antibody. Diagnosed with SLS, she was started on a tapering dose of methylprednisolone and hydroxychloroquine, along with rituximab, leading to significant improvement. Pulmonary function tests (PFTs) showed a restrictive pattern.

SLS, with a very low prevalence in SLE, can also occur in systemic sclerosis, Sjogren’s syndrome, and rheumatoid arthritis. Typical symptoms include dyspnea, pleuritic chest pain, and cough. Diagnosis involves chest radiography showing an elevated diaphragm and restrictive PFT pattern. Treatment often includes corticosteroids such as methylprednisolone and immunosuppressive agents. Rituximab has shown improvement in cases unresponsive to conventional therapy.

## Introduction

Shrinking lung syndrome (SLS) is a rare pulmonary complication primarily linked to systemic lupus erythematosus (SLE) but also observed in systemic sclerosis, Sjogren’s syndrome, and rheumatoid arthritis. Although its exact cause is unknown, theories suggest potential factors such as diaphragmatic dysfunction, pleural inflammation, and phrenic nerve paralysis.

SLS is often underdiagnosed and should be considered in patients with autoimmune diseases who present with unexplained dyspnea, reduced lung volumes, and an elevated hemidiaphragm. Diagnosis involves excluding other conditions and using chest radiography and pulmonary function tests (PFTs) to identify characteristic features.

No definitive treatment exists due to a limited understanding of SLS. Corticosteroids, particularly methylprednisolone, are commonly used, and immunosuppressive agents may also help. Recent studies indicate that rituximab, alone or with cyclophosphamide, shows promise in improving symptoms and lung function, warranting further research to confirm its effectiveness.

This article was previously presented as a meeting abstract at the Permian Basin Research Day on April 2023.

## Case presentation

A 38-year-old female with a medical history of Raynaud’s syndrome and a recent diagnosis of SLE on treatment with hydroxychloroquine 200 mg twice daily, methotrexate 10 mg every week, and folic acid 1 mg daily. She presented to the emergency room with complaints of insidious onset, gradually progressive shortness of breath, non-productive cough, and pleuritic chest pain for the past one week. Social history was significant for being a former smoker, an unquantified amount.

Admission vitals showed that she was normotensive, tachypneic with a respiratory rate of 28 cycles per minute, afebrile (temperature of 98.7°F), and saturating at 88-90% on room air. Physical examination showed a young female in mild distress due to tachypnea, unable to speak in full sentences. A lung examination showed a few fine inspiratory crackles but no pleural rub or wheeze. Mild use of accessory muscles of respiration was noted. No abnormalities were detected on cardiac, neurological, and abdominal examinations. The skin did not show any visible rash.

**Figure 1:**
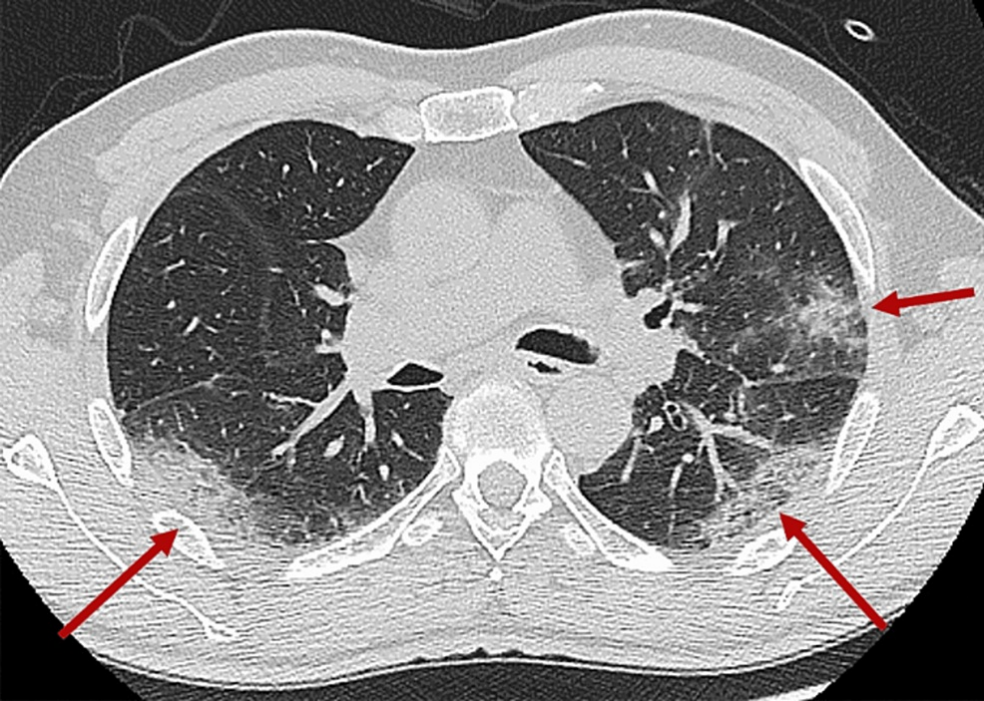
CT pulmonary angiography revealing a few bilateral interstitial infiltrates shown with red arrows.

A thorough blood work was done which showed a white blood count of 4 × 10^3^/µL, red blood cell count of 3.52 × 10^6^/µL, normocytic anemia with a hemoglobin of 9.9 g/dL, and thrombocytopenia with platelets of 68 × 10^3^/µL. A complete metabolic panel and venous lactate level were unremarkable. Inflammatory marker procalcitonin was normal and D-dimer was mildly elevated at 2.29 µg/mL. Creatinine phosphokinase, to rule out myositis, was 20 µg/L (normal = 10-120 µg/L). Arterial blood gas analysis showed primary respiratory alkalosis. Chest radiography was non-specific and revealed hazy bilateral airspace disease (Figure 1), no pulmonary embolism, and demonstrated an elevated right hemidiaphragm. An echocardiogram of the heart was also conducted which showed a left ventricular (LV) ejection fraction >75%, normal LV systolic and diastolic function, and normal right ventricular size, pressure, and function. She was initially treated with broad-spectrum antibiotics for bacterial pneumonia, along with as-needed bronchodilators and high-dose steroids with intravenous (IV) methylprednisolone for SLE increased disease activity with a Systemic Lupus Erythematosus Disease Activity Index score of 16, followed by a prednisone taper. The remaining workup was unremarkable including respiratory polymerase chain reaction (PCR), COVID-19 PCR, respiratory syncytial virus, influenza, and blood, urine, and respiratory cultures.

Inpatient pulmonary and rheumatology services were consulted. A flexible fiberoptic diagnostic bronchoscopy was done. Bacterial and fungal cultures and acid-fast bacilli were negative. Tuberculosis QuantiFERON was negative. On autoimmune workup, high titers of antinuclear antibodies 1:1,280 with speckled pattern, and positive anticardiolipin antibody, ribonucleoprotein, anti-Smith antibody, and chromatin antibody were noted. Additionally, beta-2 glycoprotein and anticardiolipin antibodies were also positive. Complement 3 was low and complement 4 was normal. Due to the above-noted decrease in lung volumes on PFTs, reduced diffusing capacity for carbon monoxide, and no evidence of interstitial disease or significant pleural disease on chest CT in the given clinical setting and the chronologic order of the disease from the time of the diagnosis, the patient was diagnosed with SLS by the agreement of the consulted rheumatology and pulmonology teams. The patient was given IV methylprednisolone 1 g/kg/day and restarted on hydroxychloroquine 200 mg twice daily. Additionally, she received rituximab 250 mg/m^2^ IV infusions and showed significant improvement with the combination. PFTs performed in the outpatient department within a week of discharge showed a restrictive pattern with reduced lung volumes, reduced diffusion capacity, and reduced respiratory muscle force.

## Discussion

SLS is a rare complication of systemic autoimmune disease that was first described in 1965 by Rosenow et al., who reported six patients with SLE who presented with symptoms of respiratory insufficiency due to a reduction in lung volume without any apparent pleuro-parenchymal disease [1]. Since then, several cases of SLS in patients with SLE have been documented. It has also been associated with rheumatoid arthritis, systemic sclerosis, Sjogren’s syndrome, scleroderma, and polymyositis, as well as in patients without any underlying autoimmune disorder [2,3]. Due to its rarity, the prevalence of SLS in other autoimmune illnesses cannot be determined; however, it is estimated to be between 0.5% and 1.53% of all lupus patients [4,5]. As in lupus, SLS affects more women than men, with a female-to-male ratio of 9:1 [6].

Within two months of receiving a lupus diagnosis, our patient was diagnosed with SLS. The delay in the lupus diagnosis may have contributed to the advancement of SLS.

Various theories have been hypothesized to explain the pathophysiology, including decreased diaphragmatic muscle thickness and diaphragmatic dysfunction secondary to pleural adhesions, pleural inflammation leading to impaired deep inspiration and eventually leading to decreased lung volumes, phrenic nerve paralysis and myopathy, and altered surfactant in the lungs. Other possible mechanisms include atelectasis and pulmonary vascular disease. Although many theories have been postulated, the exact etiology of SLS has not been proven [7-10].

The typical presentation of SLS is characterized by progressive dyspnea on exertion, pleuritic chest pain, dry cough, and fatigue, which may occur over several weeks to months, or even years. Lesser common symptoms include orthopnea and fever [11].

Physical examination may reveal decreased breath sounds, tachypnea, and signs of respiratory distress, including the use of accessory muscles of respiration. Although bi-basal crepitations may be audible due to basal atelectasis, lung auscultation is frequently normal [11].

SLS is a diagnosis of exclusion and a thorough evaluation is required to rule out alternative causes of restrictive lung disease and confirm the diagnosis of SLS. Laboratory tests may be useful to rule out infections and diagnose concomitant autoimmune diseases. Typically, the routine workup must include a complete blood count to look for leukocytosis, inflammatory markers for active lupus, and renal function tests for co-existent lupus nephritis. If not previously diagnosed, testing for a complete autoimmune profile would be appropriate. Serological profile for lupus including antinuclear antibody, complement levels, anti-double-stranded DNA, anti-Smith antibody, and anti-ribonucleoprotein. Additionally, tests for other autoimmune diseases include anti-SS-A and SS-B, rheumatoid factor, and anti-cyclic citrullinated peptide [12]. Positive antiphospholipid serology is noted in approximately two-thirds of patients, as seen in our patient. Chest imaging studies, such as plain radiography of the chest and CT scan, may demonstrate unilateral or bilateral elevation of the hemidiaphragm, along with a reduction in lung volumes. Additionally, they help exclude other parenchymal lung abnormalities and common differentials. PFT demonstrates a reduction in total lung capacity, a decreased diffusion capacity, and a restrictive pattern on spirometry [4].

Although no evidence-based guidelines for therapy have been established, treatment is primarily supportive to alleviate symptoms and improve lung function. Medium to high doses of corticosteroid followed by a taper are used invariably, either IV methylprednisolone or 0.5 to 1 mg/kg of prednisolone daily, depending on the severity of the disease [13]. Adjunctive treatments include oxygen therapy for hypoxia, inhaled beta-2 agonists, theophylline, and chest physiotherapy. Mechanical ventilation may be required in patients with respiratory failure. Immunosuppressive therapy may be used in conjunction with steroids for severe or refractory cases. Drugs that have been used in the past with variable success include azathioprine, cyclophosphamide, mycophenolate mofetil, and methotrexate. Rituximab, an anti-CD20 antibody, is a promising alternative for steroid-refractory cases. Rituximab alone or in combination with cyclophosphamide has shown improvement in symptoms and PFTs. Of the two medications, rituximab has shown positive outcomes with a decrease in the recurrence of the SLS [13]. Rarely, surgical intervention, such as diaphragmatic plication or pleurodesis, may be necessary to improve lung function.

Overall, SLS has a good prognosis, with most patients experiencing clinical improvement with therapy. However, radiological improvement is less common, seen in 57% of cases in one study [12].

## Conclusions

SLS is a rare pulmonary complication primarily associated with SLE but also observed in systemic sclerosis, Sjogren’s syndrome, and rheumatoid arthritis. Theories explaining its pathophysiology include diaphragmatic dysfunction due to pleural adhesions, phrenic nerve paralysis, and altered lung surfactant, although the exact cause remains unknown. SLS, often underdiagnosed, should be suspected in autoimmune patients with unexplained dyspnea, reduced lung volumes, and an elevated hemidiaphragm. Diagnosis involves chest radiography and PFTs, and treatment typically includes corticosteroids and immunosuppressive agents. Rituximab has shown promising results, but further research is needed due to the rarity of SLS.
